# Identification and characterization of a novel cell binding and cross-reactive region on spike protein of SARS-CoV-2

**DOI:** 10.1038/s41598-022-19886-y

**Published:** 2022-09-19

**Authors:** Hanlu Wang, Tiantian Yang, Wenhong Jiang, Meng Qin, Ziyong Sun, Wei Dai, Yongping Jiang

**Affiliations:** 1 Biopharmaceutical R&D Center, Chinese Academy of Medical Sciences, Peking Union Medical College, 88 Dongchang Road, Building M1, Suzhou, 215126 Jiangsu China; 2 Biopharmagen Corp., Suzhou, 215126 Jiangsu China; 3 Beijing Advanced Innovation Center for Soft Matter Science and Engineering, College of Life Science and Technology, Beijing University of Chemical Technology, Beijing, 100029 China; 4 Crown Bioscience, 6 Beijing West Road, Taicang, Jiangsu, 215400 China; 5 Department of Environmental Medicine, NYU Langone Health, New York, NY 10010 USA

**Keywords:** Biotechnology, Cell biology

## Abstract

Given that COVID-19 continues to wreak havoc around the world, it is imperative to search for a conserved region involved in viral infection so that effective vaccines can be developed to prevent the virus from rapid mutations. We have established a twelve-fragment library of recombinant proteins covering the entire region of spike protein of both SARS-CoV-2 and SARS-CoV from *Escherichia coli*. IgGs from murine antisera specifically against 6 spike protein fragments of SARS-CoV-2 were produced, purified, and characterized. We found that one specific IgG against the fusion process region, named COVID19-SF5, serologically cross-reacted with all twelve S-protein fragments. COVID19-SF5, with amino acid sequences from 880 to 1084, specifically bound to VERO-E6 and BEAS-2B cells, with K_d_ values of 449.1 ± 21.41 and 381.9 ± 31.53 nM, and IC_50_ values of 761.2 ± 28.2 nM and 862.4 ± 32.1 nM, respectively. In addition, COVID19-SF5 greatly enhanced binding of the full-length CHO cell-derived spike protein to the host cells in a concentration-dependent manner. Furthermore, COVID19-SF5 and its IgGs inhibited the infection of the host cells by pseudovirus. The combined data from our studies reveal that COVID19-SF5, a novel cell-binding fragment, may contain a common region(s) for mediating viral binding during infection. Our studies also provide valuable insights into how virus variants may evade host immune recognition. Significantly, the observation that the IgGs against COVID19-SF5 possesses cross reactivity to all other fragments of S protein, suggesting that it is possible to develop universal neutralizing monoclonal antibodies to curb rapid mutations of COVID-19.

## Introduction

SARS-CoV-2 has become an increasingly serious pandemic across the world due to its pathogenicity and ability to rapidly mutate, especially in the ACE-2 receptor binding motif. SARS-CoV-2 and SARS-CoV belong to the β-coronavirus genus and bind to the same cellular receptor ACE-2 during infection^1–3^. Many mutations of SARS-CoV-2 have occurred since its emergence in late 2019^4–6^. The seemingly never-ending global pandemic of COVID-19 is primarily caused by the rapid mutations of the virus, yielding variants including Delta and Omicron^7–10^. The SARS-CoV-2 spike protein is highly glycosylated, which facilitates viral infection. The glycosylation modification led to the evasion of recognition and killing of the virus by host immune systems^11–13^. Thus, it is imperative to develop more potent vaccines and other therapeutical agents in the clinic to halt the pandemic. In this report, we mapped SARS-CoV-2 and SARS-CoV spike proteins using the structure-functional approach and identified a novel cell binding and cross-actively activating region that mediated SARS-CoV-2 infection.

## Results

### Establishing a recombinant spike subunit fragment library for SARS-CoV-2 and SARS-CoV

**Figure 1:**
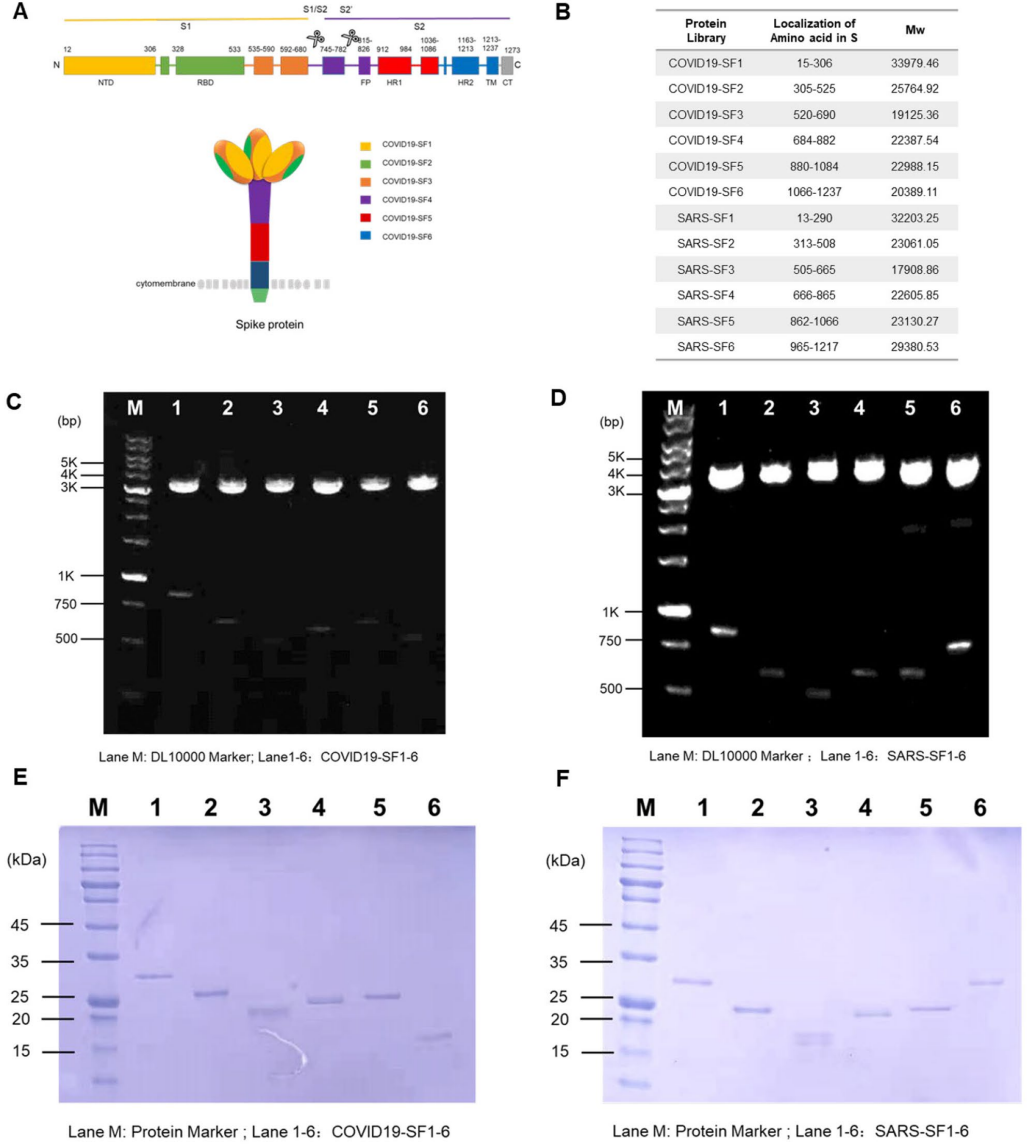
Expression and purification of recombinant spike subunit fragments of SARS-CoV-2 and SARS-CoV. (**A**) Structure features of the SARS-CoV-2 spike (S) protein and the flow chart of protein library construction were generated by Microsoft PowerPoint 2016. The listed domain boundaries are mostly defined according to NTD, N Terminal Domain; RBD, Receptor Binding Domain; FP, Fusion Peptide; HR1, Heptad Repeat 1; HR2, Heptad Repeat 2; TM, Transmembrane Domain; CT, Cytoplasmic Tail. (For visual clarity, the length of the boxes is not proportional to the real sequence length). (**B**) Amino acid location and molecular weight of the protein fragments. (**C** and **D**) Twelve protein fragments expressing plasmids of SARS-CoV-2 and SARS-CoV were constructed and verified by agarose gel electrophoresis with restriction enzymes BamH I and Hind III. M: DNA markers of known size. Lane1-6: COVID19-SF1-6 (**C**) and SARS-SF1-6 (**D**). (**E** and **F**) SDS-PAGE analysis of the protein fragments expression of SARS-CoV-2 and SARS-CoV, respectively. M: Prestained protein markers of known size. Lane1-6: COVID19-SF1-6 (**E**) and SARS-SF1-6 (**F**).

The S protein of SARS-CoV-2 consists of 1273 amino acids whereas the corresponding S protein of SARS-CoV consists of 1255 amino acids. These 2 proteins share a 78% linear homology, and divided into S1 and S2 subunits^14^. Previous studies revealed that the S1 subunit plays a major function in virus recognition and binding to host cells via the ACE-2 receptor, and that the S2 subunit is involved in the cell fusion process after initially binding^15,16^. Analysis of homologous S1 and S2 domains, in SARS-CoV-2 and SARS-CoV, reveals high linear similarities between these β-coronaviruses^17^. We have conducted studies to find if a functionally conservative amino acid fragment that shares a common antigenicity, would exist within S proteins of SARS-CoV-2 and SARS-CoV. The S1 and S2 subunits were further divided into 12 fragments, which were expressed from E. coli as shown in Fig. 1A,B. Based on amino acid structures and their known functions, these S protein fragments were named COVID19-SF1-6 and SARS-SF1-6. Among the 12 fragments, COVID19-SF1 and SARS-SF1 contained the N-terminal domains of SARS-CoV-2 and SARS-CoV, respectively. COVID19-SF2 or SARS-SF2 represents the receptor-binding domain (RBD). The remaining part of the S1 subunit was named COVID19-SF3 or SARS-SF3. The S2 subunit protein was mainly divided by size and constructed from 3 other fragments (~ 200 amino acids long). cDNAs corresponding to coding regions of 12 S protein fragments were constructed and verified by double digestion with restriction enzymes BamH I and Hind III (Fig. 1C,D). All the recombinant proteins were expressed in E. coli with 6 × His-Tag at the C-terminal and purified by Ni–NTA columns. These proteins were refolded with high yield and purity (Fig. 1E,F).

### COVID19-SF5 demonstrates strong binding ability to VERO-E6 and BEAS-2B cells

**Figure 2:**
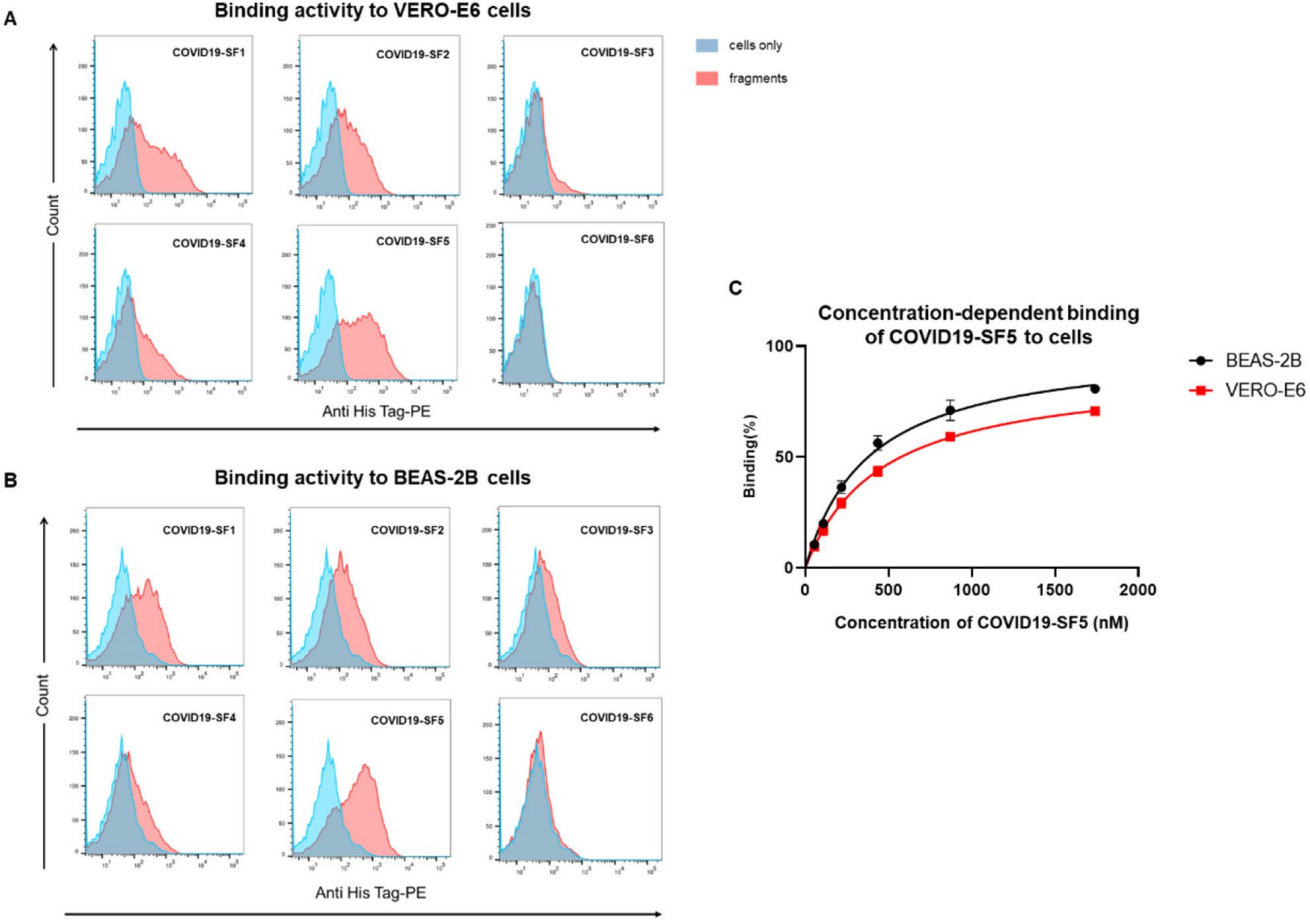
Flow cytometry and cell based-ELISA analysis of 6 fragments binding to VERO-E6 and BEAS-2B cells. (**A** and **B**) Flow cytometry was performed to assess the binding activity of 6 S protein fragments of SARS-CoV-2 to VERO-E6 cells and BEAS-2B cells. 0.25 μM His-tagged protein fragments (COVID19-SF1 ~ COVID19-SF6) were incubated with cells. The blue curves represent the cells only. The red curves represent COVID19-SF1-COVID19-SF6 binding. (**C**) Binding affinity of COVID19-SF5 to VERO-E6 and BEAS-2B cells were measured by cell based-ELISA. Cells were incubated with a series of concentrations of His-tagged COVID19-SF5 (0–40 μg/mL). The maximum binding was normalized to 100%. Mean value of 3 biological replicates mean ± SD (standard deviation) was shown. Data is a representative of 3 independent experiments.

Next, we performed flow cytometry to analyze the binding activity of 6 S protein fragments of SARS-CoV-2 to VERO-E6 and BEAS-2B host cell lines. VERO-E6 (susceptible to SARS-CoV-2 infection) and BEAS-2B (immortalized lung cell line) were incubated with 6 fragments. As shown in Fig. 2A, the COVID19-SF5 fragment strongly bound to the VERO-E6 cells and exhibited a bright fluorescence shift detected by the anti-His Tag-PE. COVID19-SF1 and COVID19-SF2 (RBD domain) also revealed some intensive binding of the cells. However, the binding fluorescence was weaker than COVID19-SF5. The binding activity of COVID19-SF3 and COVID19-SF4 was very weak and there was no detectable fluorescence shift for COVID19-SF6. In Fig. 2B, 6 protein fragments displayed a similar binding trend to BEAS-2B cells as binding to VERO-E6 cells, indicating that the 6 S protein fragments of SARS-CoV-2 showed a similar binding ability between VERO-E6 and BEAS-2B cells. The results indicate that COVID19-SF5 displays the strongest binding ability to both VERO-E6 and BEAS-2B cells.

We then further determined the binding affinity and equilibrium dissociation constants (K_d_) of COVID19-SF5 to VERO-E6 and BEAS-2B cells by a cell-based ELISA. Analysis of the fitted lines with K_d_ values of 449.1 ± 21.41 and 381.9 ± 31.53 nM for VERO-E6 and BEAS-2B cells were measured, respectively (Fig. 2C).

### Determination of specificity of COVID19-SF5 binding to VERO-E6 and BEAS-2B cells

**Figure 3:**
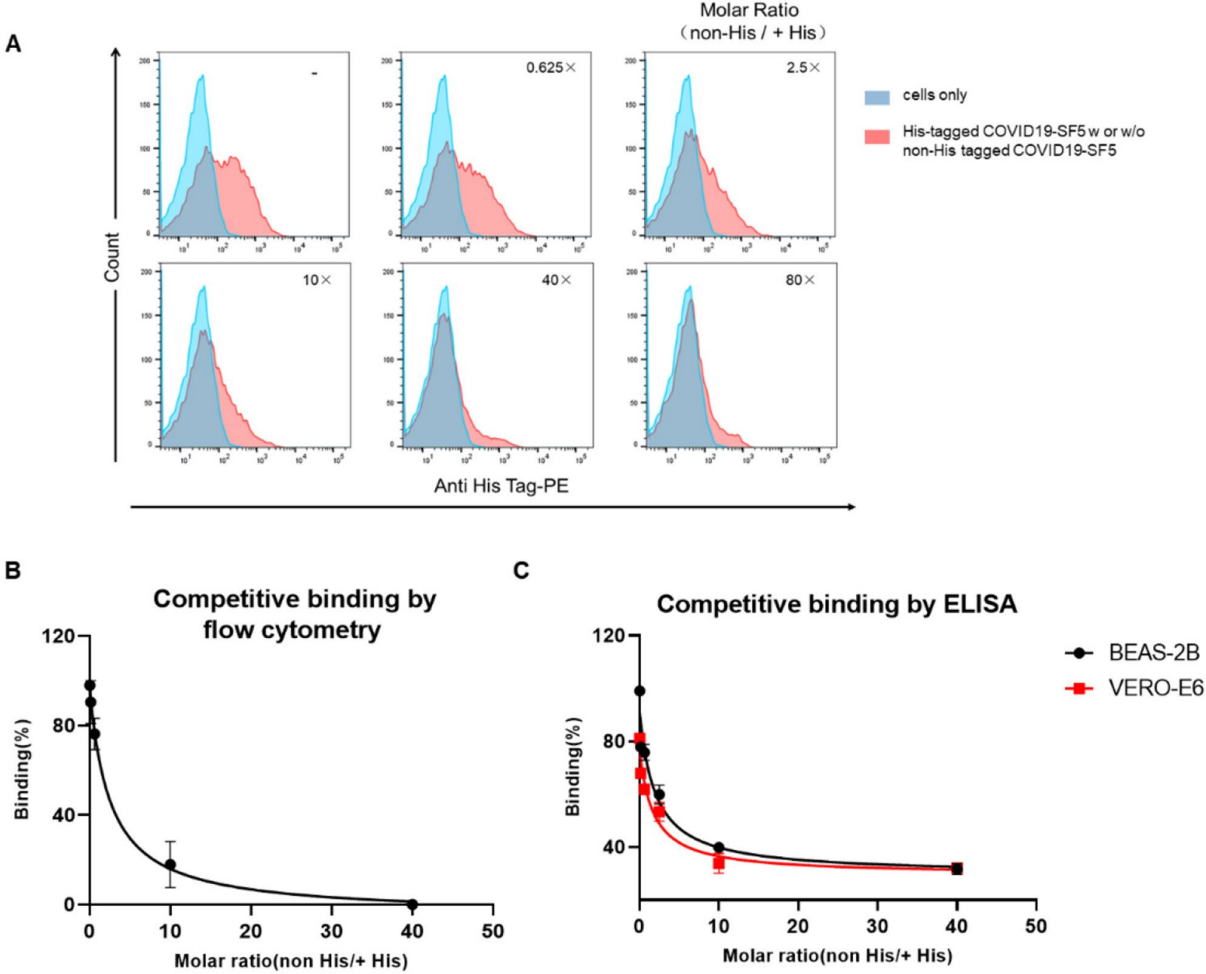
The specificity of COVID19-SF5 binding to cells by competitive binding determination. (**A** and **B**) Competitive binding of His-tagged COVID19-SF5 by non-His tagged one to VERO-E6 cells by flow cytometry. VERO-E6 cells were incubated with a series of concentrations of non-His tagged COVID19-SF5 to His-tagged one (molar ratio from 1:1 to 80:1). His-tagged protein bound to VERO-E6 cells were detected by flow cytometry. The blue curves represent the cells only. The red curves represent the His-tagged COVID19-SF5 bound to cells with a series of molar ratios. Data were a representative of 3 independent experiments. (**C**) Competitive effect of different concentrations of non-His tagged COVID19-SF5 on the binding of His-tagged COVID19-SF5 (molar ratio from 1:1 to 40:1) to VERO-E6 cells and BEAS-2B cells were detected by cell based-ELISA. The maximum bindings of His-tagged COVID19-SF5 in (**B**) and (**C**) were normalized to 100%. IC_50_ in (**B**) and (**C**) was calculated by fitting to a sigmoidal dose–response curve with mean ± SD (standard deviation) of triplicated independent assessments.

The binding specificity of COVID19-SF5 to VERO-E6 cells was further examined by determining the ability of 6 × His-tagged COVID19-SF5 to competitively bound to VERO-E6 cells in the presence of a series of concentrations of non-His tagged COVID19-SF5 with a molar excess up to 80-folds. As shown in Fig. 3A, the His-tagged protein binding to VERO-E6 cells was decreased gradually as non-His tagged COVID19-SF5 increased, and was completely inhibited at the 40-fold molar excess of non-His tagged COVID19-SF5. We then performed the competitive binding experiments 3 times independently and calculated IC_50_ values of 1.1 ± 0.38 μM for the non-His tagged COVID19-SF5 binding to VERO-E6 cells (Fig. 3B). Furthermore, we have conducted the competitive binding study using a cell based-ELISA approach. As shown in Fig. 3C, the non-His tagged COVID19-SF5 inhibited His-tagged protein bound to VERO-E6 and BEAS-2B cells with IC_50_ values of 761.2 ± 28.2 nM and 862.4 ± 32.1 nM, respectively.

These results confirm that COVID19-SF5 specifically binds to both VERO-E6 and BEAS-2B cells and that the His-tag modification does not alter the binding activities of COVID19-SF5 to the cells.

### The production of mouse anti-sera against six S subunit fragments of SARS-CoV-2

To identify common antigenic epitopes between SARS-CoV-2 and SARS-CoV, BALB/c mice were immunized by 6 SARS-CoV-2 fragments with adjuvants as described previously by our group^18^. IgGs from the antisera were then purified by the MabSelect™ PrismA affinity column. Six pooled IgGs were collected and standardized at concentration of 50 μg/mL. Purified IgGs against 6 S subunit fragments of SARS-CoV-2 were titrated by ELISA. Strong immunoreactivities were detected for all 6 fragments of SARS-CoV-2 (from fragments COVID19-SF1 to 6), with titers from 1:3200 to 1:12,800.

### IgGs of COVID19-SF5 cross-reacting with 12 protein fragments of SARS-CoV-2 and SARS-CoV by ELISA and immunoblotting

**Figure 4:**
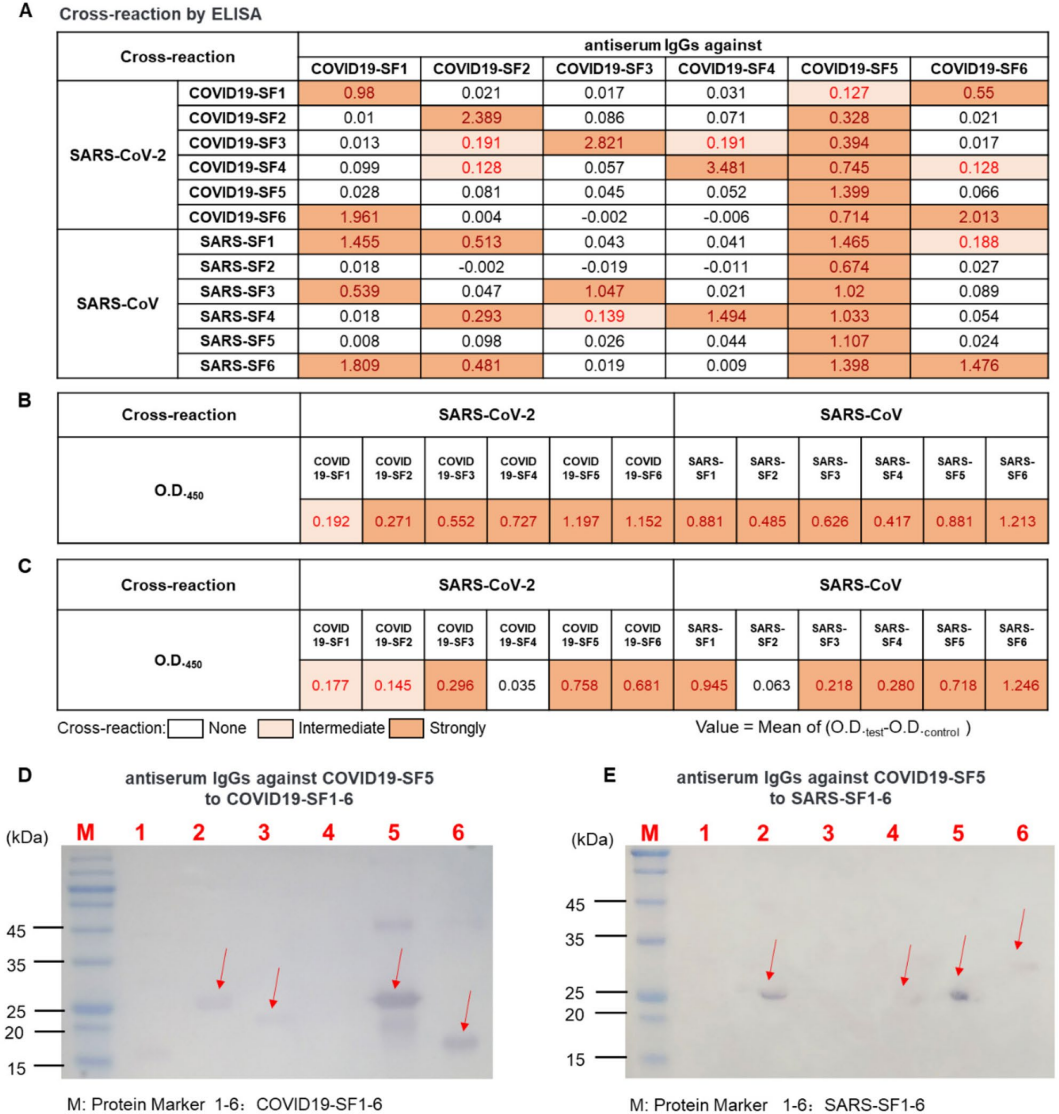
Cross reaction of the IgGs against COVID19-SF5 to 12 fragments. (**A**) ELISA testing of the IgGs (0.05 μg/mL) against 6 protein fragments of SARS-CoV-2 with the 12 protein fragments (10 pmol/well) of SARS-CoV-2 and SARS-CoV. (**B** and **C**) ELISA testing of the IgGs (0.05 μg/mL) against COVID19-SF5 with the 12 protein fragments (10 pmol/well) of SARS-CoV-2 and SARS-CoV. Antiserum was obtained after 3 (**B**) or 6 (**C**) months from the first immunization. Deep pink indicates a strong reaction (O.D._test_ − O.D._control_ > 0.2), light pink indicates an intermediate reaction (0.2 > O.D._test_ − O.D._control_ > 0.1), and white indicates no reaction (O.D._test_ − O.D._control_ < 0.1). Mean values of 3 biological replicates are shown. (**D** and **E**) Immunoblotting of anti-serum (0.2 μg/mL) against COVID19-SF5 with 12 fragments (1 μg) of SARS-CoV-2 (**D**) and SARS-CoV (**E**).

Cross-reactivities of IgGs against each of COVID19-SF1-6 to 12 S protein fragments of SARS-CoV-2 and SARS-CoV were further analyzed. As shown in Fig. 4A, IgGs from COVID19-SF5 not only reacted strongly against its own fragment, but also intensively cross reacted with all other 5 fragments of SARS-CoV-2. Interestingly, IgGs against COVID19-SF5 also strongly cross-reacted with all 6 recombinant S protein subunit fragments of SARS-CoV in ELISA. Moreover, strong cross-reactivity remained detectable with all twelve protein fragments 3 months after the first immunization (Fig. 4B). Over time, COVID19-SF5 antiserum reacted with most of the subunit fragments, although the reactivity gradually diminished. In fact, the antiserum no longer reacted with COVID19-SF4 and SARS-SF2 6 months after the first immunization (Fig. 4C). These cross-reaction activities indicate that COVID19-SF5 shares common antigenicity with all twelve S protein fragments of SARS-CoV-2 and SARS-CoV.

In order to confirm the cross-reactions observed from ELISA, immunoblotting was performed by using COVID19-SF5 IgGs on all 12 recombinant protein fragments of SARS-CoV-2 and SARS-CoV. In Fig. 4D,E the immunoreactive band(s) were observed with fragments 2, 3, 5, 6 of SARS-CoV-2 and 2, 4, 5, 6 of SARS-CoV; no immunoreactive band(s) was observed with the remaining fragments of SARS-CoV-2 and SARS-CoV. This can be explained by the fact that denatured protein fragments were used in immunoblotting whereas native proteins were used in ELISA. Overall, the results from ELISA and immunoblotting were consistent, and IgGs against COVID19-SF5 strongly cross-react with all these protein fragments.

### IgGs against COVID19-SF5 inhibiting COVID19-SF5 binding to VERO-E6 cell

**Figure 5:**
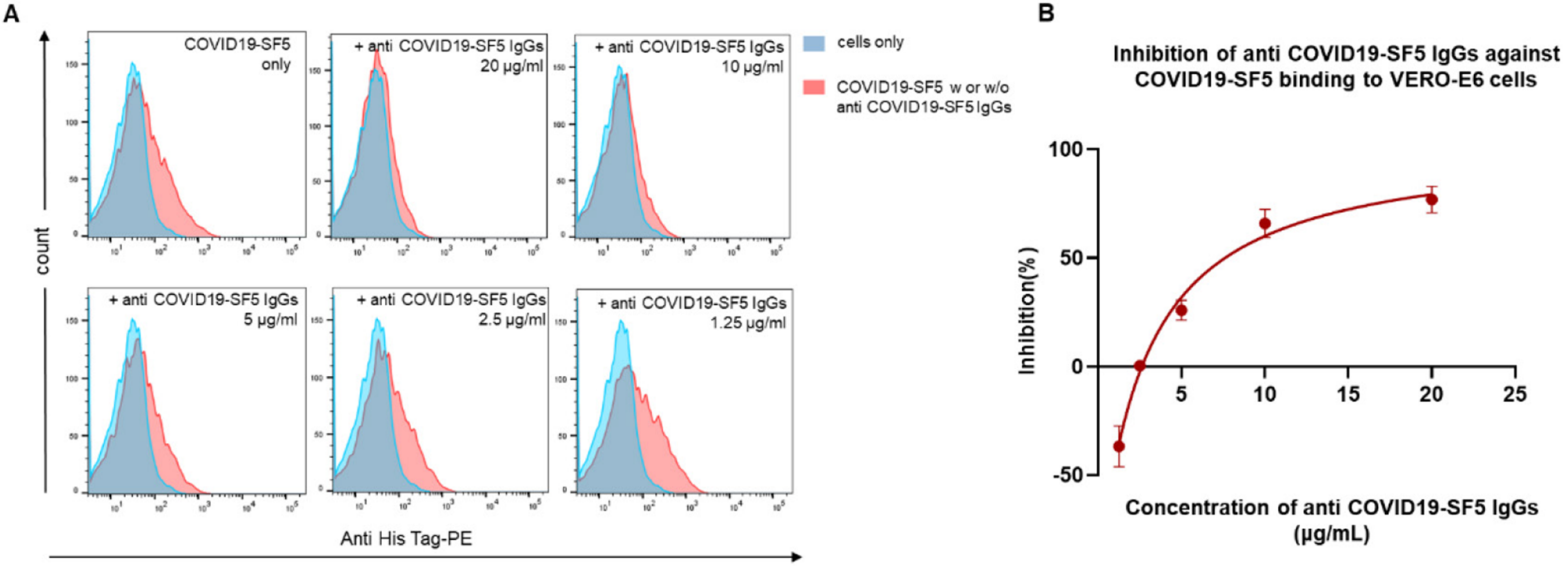
The inhibition of anti-serum antibodies of COVID19-SF5 on COVID19-SF5 binding to cells. (**A** and **B**) Flow cytometry to detect the effect of IgGs against COVID19-SF5 (0–20 μg/mL) on COVID19-SF5 (0.25 μM) binding to VERO-E6 cells. The blue curves represent the blank control (cells only). The red curves represent the binding of COVID19-SF5 itself or with various concentrations (1.25–20 μg/mL) of anti-serum antibodies against COVID19-SF5. A representative curve of concentration-dependent inhibition was shown in (**B**).

To further characterize the effect of anti-serum against COVID19-SF5 on COVID19-SF5’s binding to the cells, flow cytometry was conducted. After co-incubation of specific IgGs with COVID19-SF5 and VERO-E6 cell, COVID19-SF5 binding levels were then analyzed. As shown in Fig. 5A,B, IgGs against COVID19-SF5 inhibited the fragment binding to the cell in a concentration-dependent manner.

### Impacting of fragments and their anti-sera of SARS-CoV-2 on full-length S protein binding to the cells

**Figure 6:**
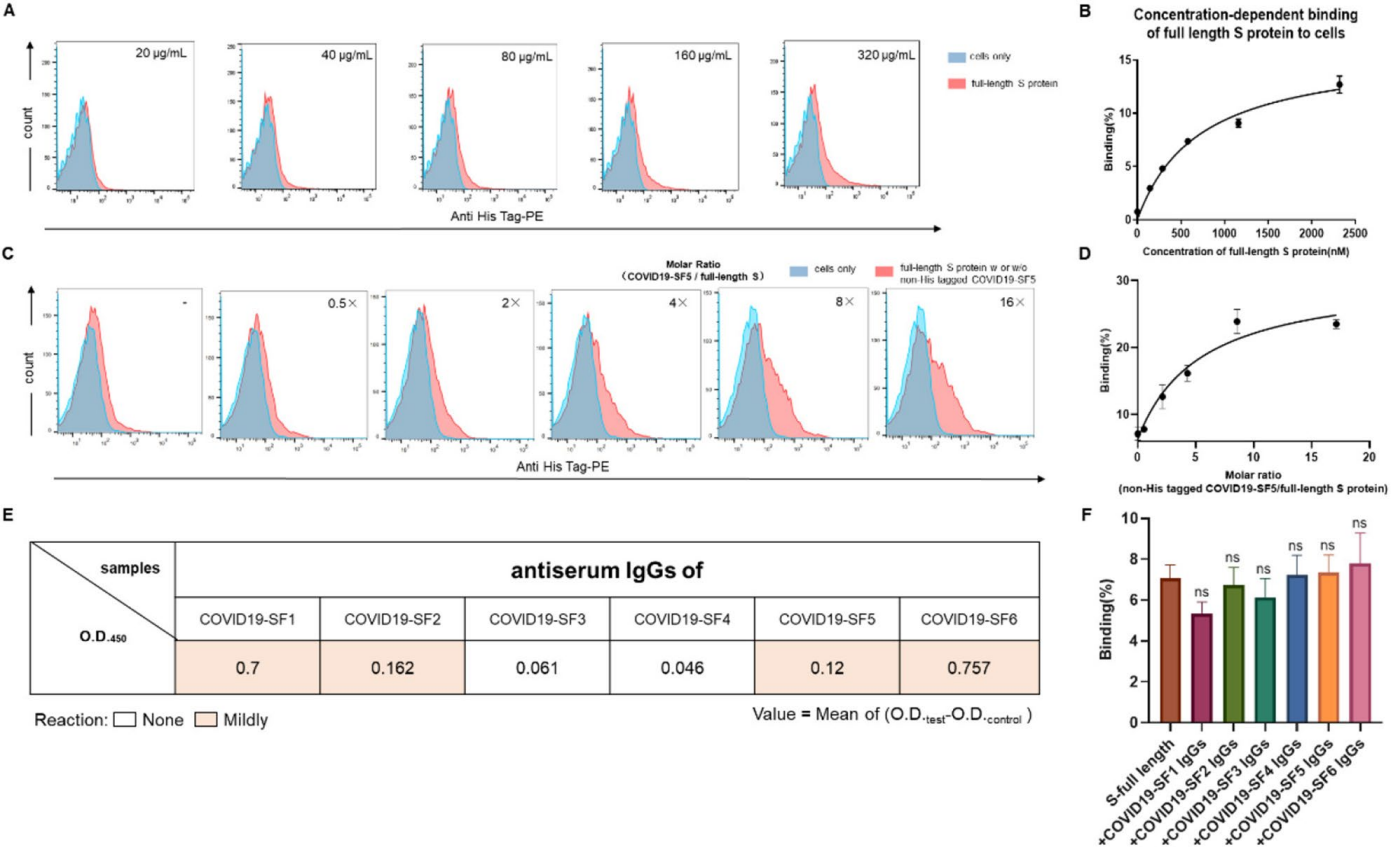
The effect of COVID19-SF5 and S protein fragment anti-serum antibodies on the binding of full-length glycosylated S protein to the cells. (**A** and **B**) A concentration-dependent binding of the full-length S protein to VERO-E6 cells. VERO-E6 cells were incubated with a series of concentrations (0–320 μg/mL) of His-tagged full-length spike protein. The blue curves represent the cells only. The red curves represent the full-length S protein binding to the cells. The quantified data were shown in the right panel. Mean values of 3 biological replicates ± SD (standard deviation) were shown. (**C** and **D**) Flow cytometry characterizing the enhancement of non-His tagged COVID19-SF5 on full-length S protein binding to VERO-E6 cells. VERO-E6 cells were incubated with a series of concentrations of non-His tagged COVID19-SF5 to His-tagged full-length spike protein (molar ratio from 0.5 to 16: 1), and His-tagged full-length spike protein bound to VERO-E6 cells were detected by flow cytometry. The blue curves represent the cells only. The red curves represent the full-length S protein itself or with non-His tagged COVID19-SF5. The quantified representative was shown in the right panel. Mean values of 3 biological replicates ± SD (standard deviation) were shown. Data were a representative of 3 independent experiments. (**E**) Reactions of the anti-serum antibodies (0.05 μg/mL) against 6 protein fragments with full-length S protein (10 μg/mL) by ELISA characterization. Light pink indicates intermediate reaction (1 > O.D._test_ − O.D._control_ > 0.1), and white indicates no reaction (O.D._test_ − O.D._control_ < 0.1). Mean values of 3 biological replicates were shown. (**F**) Flow cytometry determination of the effects of 6 anti-serum antibodies on the binding of full-length S protein to VERO-E6 cells. Cells were incubated with His-tagged full-length spike protein (35 μg/mL) and anti-serum IgGs (10 μg/mL) against 6 protein fragments. Mean values of 3 biological replicates ± SD (standard deviation) were shown (one-way ANOVA).

His-tagged full-length S protein, expressed from the CHO cell, was further employed to determine the specific binding of the protein and antibodies we produced. Firstly, flow cytometry was performed to assess the binding activity of full-length S protein of SARS-CoV-2 to host cell lines. As shown in Fig. 6A,B, full-length S protein bound to cells in a concentration-dependent manner. Analysis of the fitted lines revealed K_d_ values of 713.2 ± 111.6 nM for VERO-E6 cells. The binding ability of full-length S protein to VERO-E6 was much lower than that of COVID19-SF5. One possible reason for this is that the full-length S protein expressed from CHO cell was fully glycosylated, which potentially masks some binding sites in the cells^19^.

Secondly, to further investigate the effect of COVID19-SF5 on full-length S protein cell binding, various concentration of non-His tagged COVID19-SF5 (0.5–16-fold molar excess) was added to determine the influence on the full-length S protein to VERO-E6 cell. As shown in Fig. 6C,D, non-His tagged COVID19-SF5 significantly enhanced full-length S protein binding to VERO-E6 cells in a concentration-dependent manner. These results indicate that COVID19-SF5, a domain not exposed in the full-length glycosylated S protein, enhances the binding of full-length S protein to the cell, which may facilitate virus infection and fusion process after the initial ACE-2 binding.

Thirdly, serological reactions of the 6 anti-sera to the full-length glycosylated S protein were detected by ELISA, as shown in Fig. 6E. Among them, the full-length glycosylated S protein reacted with anti-serum against fragments COVID19-SF1, 2, 5, and 6, but not with anti-sera from COVID19-SF3 and 4. In addition, the antibodies on the binding of full-length S protein to VERO-E6 cells were also tested by flow cytometry. As shown in Fig. 6F, none of the IgGs displayed an apparent effect on full-length S protein binding to VERO-E6 cells, indicating occlusion of S protein by glycosylation and conformation.

### Inhibition of pseudovirus infection by S protein fragments and anti-serum antibodies

**Figure 7:**
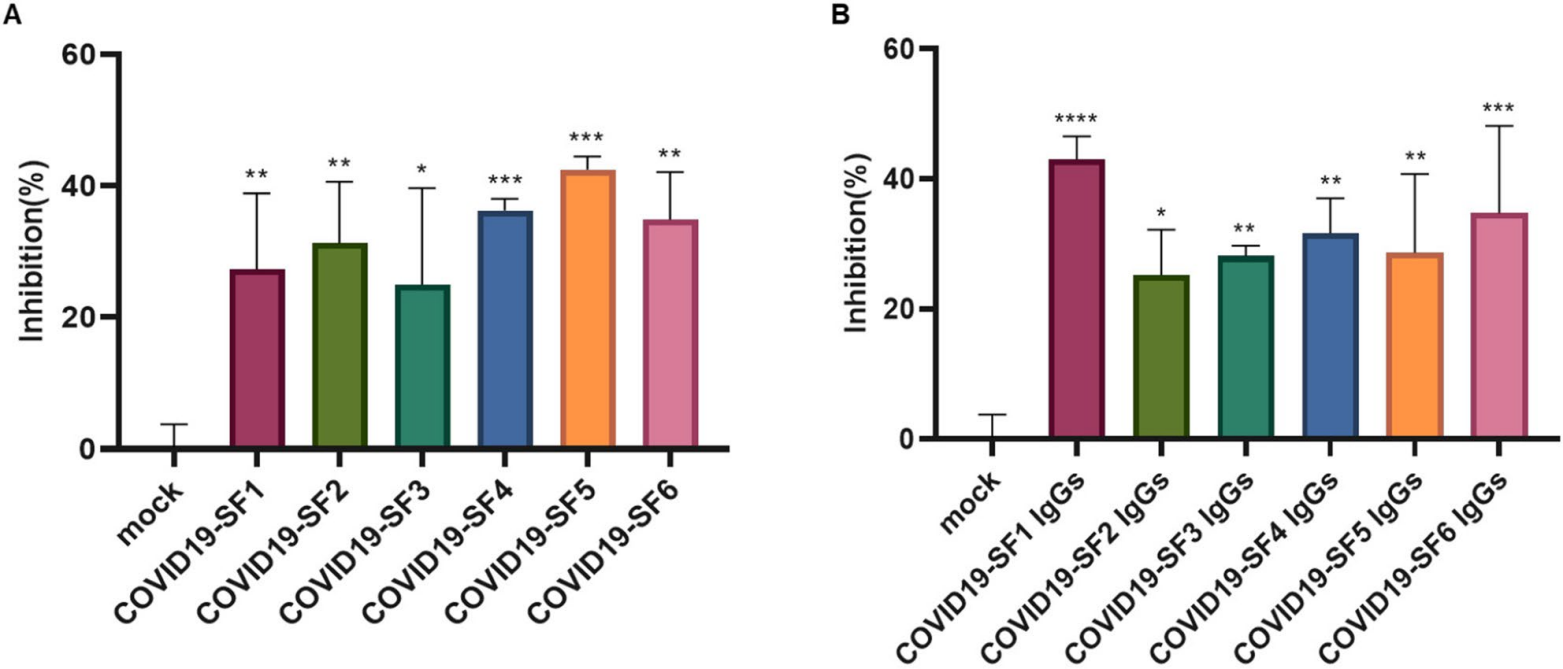
The effect of S protein fragments and their antiserum on pseudovirus infection to the cells. (**A**) Six protein fragments (1 μM) of SARS-CoV-2 were incubated with pseudovirus (650 TCID_50_) and hACE2-293T cells, and infection was analyzed by the luciferase system. (**B**) IgGs (10 μg/mL) against 6 protein fragments of SARS-CoV-2 were incubated with pseudovirus (650 TCID_50_) and hACE2-293T cells, and infection was analyzed by the luciferase system. The luciferase in the cells was quantified by a microplate luminometer. The Y-axis of the graphs represents the mean percentage of inhibition of virus yield. Inhibition rate was normalized based on uninfected cells as 100% inhibition and cells infected with pseudovirus only (mock) as 0% inhibition of the luciferase activity. Values were shown as mean of triplicates ± SD; *p < 0.05, **p < 0.01, ***p < 0.001, ****p < 0.0001 by one-way ANOVA.

For neutralization analysis, 6 protein fragments, COVID19-SF1 to COVID19-SF6, and their 6 corresponding IgGs were used to characterize the neutralization ability of pseudovirus infection in 96-well plates. As shown in Fig. 7A, the 6 recombinant fragment proteins inhibited SARS-CoV-2 pseudovirus infection with infection rate from about 20–50%. Among them, COVID19-SF5 displayed the strongest inhibition of pseudovirus infection to the cells. In addition, the 6 anti-sera also showed some neutralization effect on SARS-CoV-2 pseudovirus infection of the cells (Fig. 7B).

Collectively, our data indicate that both proteins and specific IgGs were able to neutralize pseudovirus, thus preventing the infection of host cells although the effect varied between IgGs and fragments.

## Discussion

The ongoing global COVID-19 pandemic is surging due to continuous mutations of SARS-CoV-2, which had led to the emergence and spread of Delta and Omicron variants^20–22^. The COVID-19 virus spike protein plays a crucial role during infection, either in the ACE2-receptor binding or subsequent fusing with the cell membrane prior to the injection of virus nuclei acid into the host cells^16,23^. In addition, the spike protein is a major element that activates host humoral and cellular immunity for preventing the virus from infection^24^. Spike protein is heavily modified by glycosylation, which may shield the virus from recognition by the host immune system during viral infection^25^. The binding of spike protein to ACE-2 cellular receptors is considered the most important initial step during infection by SARS-CoV-2. The RBD region binding to ACE-2 is the major target region in developing effective vaccines and potent virus-neutralizing antibodies^26–28^. However, some neutralizing mAbs targeting S protein of SARS-CoV-2’s derived from COVID-19 patients do not react with the ACE-2 receptor binding motif^29,30^.

At present, the highly transmissible Omicron variant is spreading rapidly across the world, causing new infections, reinfections, and breakthrough infections among fully vaccinated people^31,32^. There may be an alternative infection pathway for mutant RBD-ACE-2 virus variants to infect host cells and some other functional or active regions on the SARS-CoV-2 spike protein to be further identified. In this report, we used peptide mapping of all regions of the S protein to assess the structural and functional relationship in cell binding, serological, and pseudovirus infection assays. Given that each antibody from the pool of polyclonal antibodies could serologically overlap with other(s) due to a common epitope sequence(s) and/or that the epitopes may share similar features involved in their structural folding, our data show that the new cell binding region of COVID19-SF5 with cross-reactive antigenicity may comprise a conserved functional fragment required for the virus fusion process during infection. Because the region COVID19-SF5 is embedded and hidden by trimer spike proteins or glycosylation, the host immune system may fail to recognize the fragment as a foreign invading protein during virus initial infection. As S protein initially interacts with the cells via ACE-2 binding, it may change the conformation or deglycosylation of the S protein to expose COVID19-SF5 region for enhanced binding to the cells. This binding may make cell surface more accessible to other S protein binding regions, such as region COVID19-SF1 and COVID19-SF2. Consequently, binding of COVID19-SF5 to the cell enhances the full-length S protein binding to the cells as shown in Fig. 6C,D. This enhanced binding may help the virus become more transmissible, like in the case of the Omicron variant that easily escapes humoral immunity and remains infectious to fully vaccinated people^33–35^.

In summary, a new cell-binding region (COVID19-SF5) on spike protein of SARS-CoV-2 has been identified and characterized. The binding of COVID19-SF5 to the cell can greatly enhance the association of glycosylated full-length S protein with the host. In addition, a specific IgG against COVID19-SF5 cross-reacts with all fragments of SARS-CoV-2 and SARS-CoV. Based on these findings, we propose that an effective COVID-19 vaccine can be developed by combining only fragments COVID19-SF2 (RBD) and COVID19-SF5 when we design specific mRNA vaccines and the vaccines of other technologies. Further, COVID19-SF5 and its specific IgG inhibit pseudovirus infection to the host cells, strongly suggesting that conserved binding sites (epitopes), besides the RBD-ACE-2 site, may exist in these regions. Thus, a potent universal neutralizing monoclonal antibody (or antibodies) can be developed to combat COVID-19 and its emerging variants.

As it is now understood, the prefusion highly glycosylated spike exists in various states or configurations that masks immunodominant regions. The identification of a highly occluded region of spike being important as an immunogen is intriguing and does require further scrutiny. That peptide SF5 enhances recognition of full-length spike does support our conclusion that its epitopes are somewhat cryptic. However, it is intriguing that it enhances rather than inhibits spike recognition. Our studies suggest that this activity likely occurs after ACE2 binding, it might be worth suggesting potential mechanisms. Does SF5 peptide 'open' up Spike thus changing the configuration to represent one that is more efficient at undergoing fusion? To better understanding the hypotheses, we are currently conducting configuration studies with full-length spike binding with or without SF5 in our separate study.

## Materials and methods

### Oligonucleotides

The plasmids containing the spike gene of SARS-CoV-2 and SARS-CoV were purchased from Sangon Biotech (Shanghai, China) and Sino Biological Inc. (Beijing, China), respectively. Designed primers for gene amplification were synthesized by Sangon Biotech Co., Ltd. and were listed in Supplemental Table 1.

### Cell lines and pseudotyped virus

VERO-E6 and BEAS-2B cell lines were obtained from Cell Resource Center of Shanghai Institutes for Biological Sciences. hACE2-293T were purchased from Delivectory Biosciences Inc. (Beijing, China). VERO-E6, BEAS-2B and hACE2-293T were cultured in DMEM medium supplemented with 10% (v/v) fetal bovine serum (FBS), 100 U/mL penicillin, and 100 mg/mL streptomycin in an incubator (Thermo Fisher Scientific, USA) at 37 °C, 5% CO_2_. Cells were sub-cultured routinely. SARS-CoV-2 pseudovirus expressing luciferase were purchased from Delivectory Biosciences Inc. (Beijing, China).

### Animals and ethics statement

Female BALB/c mice were purchased from SLRC Laboratory Animal Center (Shanghai, China). All animal studies were performed in accordance with the Guide for Care and Use of Laboratory Animals, Soochow University. The animal experimental protocols were approved by the Soochow University Institutional Animal Care and Use Committee (IACUC Permit Number SYXK (Su) 2020-0210). Our report follows the recommendations in the ARRIVE (Animal Research: Reporting of In Vivo Experiments) guidelines. At the end of the experiments, euthanasia was performed using ketamine/xylazine 100/10 mg/kg intraperitoneally.

### Plasmid construction

The sequences encoding COVID19-SF1 (amino acids 15-306), COVID19-SF2 (amino acids 305-525), COVID19-SF3 (amino acids 520-690), COVID19-SF4 (amino acids 684-882), COVID19-SF5 (amino acids 880-1084) and COVID19-SF6 (amino acids 1066-1237) of SARS-CoV-2, and SARS-SF1 (amino acids 15-292), SARS-SF2 (amino acids 315-510), SARS-SF3 (amino acids 507-667), SARS-SF4 (amino acids 668–867), SARS-SF5 (amino acids 864-1068) and SARS-SF6 (amino acids 967-1219) of SARS-CoV were amplified with listed primers, fused with 6 × His tag at the C-terminal and restriction enzyme cutting site. Digested with restriction enzymes BamH I and Hind III, the amplified genes were cloned into pQE-3 expression vector. These plasmids expressing S subunit fragments were transformed into *E. coli* M15. The DNA encoding the extracellular domain sequence of spike protein (1-1208) with proline substitutions at residues 986 and 987, a “GSAS” substitution at residues 682–685, and a C-terminal 8 × His Tag was synthesized and cloned into the expression vector pcDNA3.4^36^.

### Protein expression and purification

The protein fragments were expressed in a form as inclusion body. The inclusion bodies were collected from IPTG induced bacteria lysates and dissolved with Guanidine Hydrochloride (6 M). Ni–NTA Purification System was employed to purify poly histidine-containing proteins as previously described^37^. The purified proteins were then diluted with a sodium acetate buffer containing 8 Mol of Urea (PH 5.4) to 0.2 mg/mL, and the refolding protein was obtained by a series of dialysis procedure with optimized buffer conditions at 4 °C.

For the extracellular domain of the full-length spike protein (1-1208) was produced by transfecting 1 Liter ExpiCHO-S™ Cells at a density of 6 × 10^6^ cells/mL. After cultured at 37 °C for a week, the supernatant was harvested and purified with Ni–NTA chromatography.

For non-His tagged protein, the dissolved inclusion body was diluted and underwent a series of dialysis procedure. The refolding proteins were further purified by CM-Sepharose and Supersex-75 chromatography followed the procedures published before^38^.

### Flow cytometry analysis of protein fragments binding to cells

VERO-E6 and BEAS-2B cells were digested with 0.25% trypsin. Next, 1.5 × 10^5^ cells were incubated with His-tagged protein fragments at 0.25 μM for 1 h at 37 °C, except for the control groups. The cells were then washed and stained with PE anti-His Tag antibody (Biolegend, San Diego, USA) for 1 h at 4 °C. The cells were further washed and resuspended, subjected to flow cytometry analysis. A total of 20,000 events were acquired for each sample using the BD FACSVerse™ system (Becton Dickinson, Franklin Lakes, NJ)^39–42^. Data were analyzed by FlowJo software (Version 10.5.3, Tree Star Software; CA, USA) as described previously^43^.

### Concentration-dependent binding of COVID19-SF5 by cell-based ELISA

96 microtiter-plates were seeded with VERO-E6 or BEAS-2B cells at 20,000 cells/well. The cells were washed by PBS and fixed with 4% formaldehyde 1 day later. 3% H_2_O_2_ were added to quench endogenous peroxidase. Cells were then blocked with 1% BSA in PBS for 1 h at 37 °C. Serially concentrations (0–40 μg/mL) of COVID19-SF5 were added and incubated with the cells for 2 h at 37 °C. Horseradish peroxidase (HRP) anti-His Tag antibody (1:20,000, Biolegend, San Diego, USA) were added after washing. After another 3 times washing, the plates were developed with 3,3′,5,5′-tetramethylbiphenyldiamine (TMB) for 15 min. The reactions were stopped by the addition of 2 M H_2_SO_4_ stop solution. The absorbance was measured on a microplate reader at 450 nm.

### Specificity of COVID19-SF5 binding by flow cytometry analysis

Flow cytometry was performed to determine the competitive binding of the His-tagged COVID19-SF5 by non-His tagged one. The 1.5 × 10^5^ cells were incubated with 10 μg/mL His-tagged COVID19-SF5 and a series of concentrations of non-His tagged COVID19-SF5 (molar ratio from 0.625 to 80 excess) for 1 h at 37 °C. All the other steps were same to that described for the flow cytometry analysis. Nonlinear regression analysis curve was fitted by GraphPad Prism 7.0 and IC_50_ value was calculated^44^.

### Specificity of COVID19-SF5 binding by cell-based ELISA

VERO-E6 or BEAS-2B cells were seeded and treated as described for cell-based ELISA. Subsequent to blocking, cells were incubated with 10 μg/mL His-tagged COVID19-SF5 and a series of concentrations of non-His tagged COVID19-SF5 (molar ratio from 0.625 to 40 excess) for 1 h at 37 °C. All the other steps were the same as above. Specific binding was calculated by subtracting nonspecific binding, as determined by the addition of a 40-fold excess of non-His tagged COVID19-SF5. Nonlinear regression analysis curve was fitted by GraphPad Prism 7.0 and IC_50_ value was calculated.

### Mouse immunization, sample collection, and purification

BALB/c mice aged 6–8 weeks (n = 3) were immunized by intramuscular (IM) injection with 2 doses spaced 14 days apart (days 0 and 14) containing 0.1 mg of each fragment of SARS-CoV-2 and Freund's complete or incomplete adjuvant^18^. The mice received a boost dose with 0.1 mg of protein fragments without adjuvant on Day 28. Serum was collected 2 days after the last injection. The antiserum of mice immunized with COVID19-SF5 was continuously collected on day 90 and day 180 for cross-reactive measurement.

Serum IgGs against 6 fragments were then purified by MabSelect™ PrismA (GE Healthcare) according to the manufacturer’s instructions. The concentration of the purified IgG was determined using a standard Bio-Rad Bradford method^45^.

### Titer determination and cross-reaction of serum IgGs by ELISA

The 96-well microtiter plates (Costar) were coated with 50 μL of fragments at 2 μg/mL overnight at 4 °C. The plates were washed with PBS-T and blocked with PBS-T containing 1% BSA. Subsequently, IgGs at series of dilutions (1:100–1:25,600) were added to the plates for 2 h incubation at 37 °C. Followed by washing, goat anti-mouse IgG HRP-conjugated antibody (Sangon Biotech, 1:10,000) was added and incubated for 1 h at 37 °C Plates were then washed 3 times and the color reaction was developed with 3,3′,5,5′-tetramethylbiphenyldiamine (TMB) substrate. After 15 min incubation, the reaction was stopped by 2 M H_2_SO_4_ stop solution. The absorbances at 450 nm were measured on a Thermo microplate reader^46^.

For cross-reaction determination, plates were coated with the fragments at same molar quantities (10 pmol/well). The IgGs were added at 0.05 μg/mL. All the other steps were the same as above.

To determine the reaction of IgGs to the full-length spike protein, plates were coated with the full-length spike at 10 μg/mL overnight at 4 °C. The IgGs against 6 fragment proteins were added at 0.05 μg/mL. All the other steps were the same as the cross-reaction determination as above.

### Immunoblot analysis

Protein fragments were separated on the denaturing sodium dodecyl sulfate (SDS)-polyacrylamide (12%, w/v) gel before transferred to the polyvinylidene difluoride (PVDF) membrane. PVDF membranes were then blocked with 4% BSA in TBS overnight at 4 °C. After washing, PVDF membranes were incubated with serum IgG against COVID19-SF5 for 2 h at room temperature. Alkaline phosphatase (AP) conjugated goat anti-mouse IgG was used as secondary antibody. After 1 h incubation, the immunoreactive bands were detected by BCIP/NBT alkaline phosphatase color reagent kit (Absin, China) according to the manufacturer’s instructions and the modified procedure from our lab^47,48^.

### Inhibition of the COVID19-SF5 anti-serum antibodies to COVID19-SF5 binding to cells

1.5 × 10^5^ cells were incubated with 0.25 μM His-tagged COVID19-SF5 and serially concentrations (1.25–20 μg/mL) of IgGs against COVID19-SF5 for 1 h at 37 °C. All the other steps were same to that described for the flow cytometry analysis.

### Concentration-dependent binding of full-length spike to cells

The 1.5 × 10^5^ cells were incubated with a concentration series (0–320 μg/mL) of His-tagged full-length spike protein in 50 μL FACS buffer for 2 h at 37 °C. The cells were then washed and stained with PE anti-His Tag antibody (BioLegend, San Diego, CA) according to the manufacturer’s protocol. The cells were subjected to flow cytometry analysis as set forth. The binding frequency of each sample was determined to calculate the dissociation constant (K_d_) between the protein and cells by non-linear fitting^49,50^.

### Effect of the antibodies and COVID19-SF5 on full-length spike binding to cells

Cells were incubated with His-tagged full-length spike protein and anti-serum IgGs against 6 protein fragments or non-His tagged COVID19-SF5, respectively. The final concentration of full-length spike protein was at 35 μg/mL, and at 10 μg/mL of antibodies in the mixture. A series of concentration of non-His tagged COVID19-SF5 (molar ratio from 0.5 to 16 excess) was added in the COVID19-SF5-treated group. All the staining and detection steps were the same as described previously for flow cytometry.

### Neutralization of pseudovirus infection assay

The pseudovirus neutralization experiment was performed as described previously^51^. hACE2-293T cells were seeded at the 96 well plates with 2 × 10^4^/well. The final concentration of 6 proteins is configured as 1 μM. The final concentration of IgGs are configured as 10 μg/mL. These samples were mixed with a certain amount (650 TCID_50_/well) of pseudotyped virus. The mixture was respectively added into the cells and incubated for 48 h. The expression of luciferase was measured by a multifunctional microplate reader (Molecular Devices, USA) according to the manufacturer’s protocol to obtain the antiviral activity of the samples. The cell control with only cells and the virus control with virus and cells (mock) are set up in each plate. Three parallel experiments were set in each group. The inhibition rate of protein samples on virus was further calculated.

### Statistical analysis

Results are presented as the mean ± standard deviation of at least 3 independent experiments with duplicate samples. Statistical differences were evaluated using one-way analysis of variance unless otherwise indicated. *p* < 0.05 was considered as statistically significant. Graphs were generated by GraphPad Prism (version 7; GraphPad, La Jolla, CA, USA), Microsoft PowerPoint and Microsoft Excel.

## Supplementary Information

Supplementary Information.

## Data Availability

Publicly available datasets employed in this study include the SARS-CoV-2 S protein reference sequence (NCBI accession MN908947.3) and the SARS-CoV S protein reference sequence (NCBI Accession AAP13567.1).The uncropped gels for Figs. 1C–F, 4D,E were shown in Supplementary Figs. 1, 2, 3, 4, 5 and 6 respectively. All other data supporting the conclusions of the current study are available from the corresponding author upon reasonable request.
