# Supplementary material for: Identification and characterization of a novel cell binding and cross-reactive region on spike protein of SARS-CoV-2
Source: Sci Rep. 2022 Sep 19;12:15668. doi: 10.1038/s41598-022-19886-y (PMC9484712; doi:10.1038/s41598-022-19886-y)
Supplement: Supplementary file 1 — Supplementary Information. [file 41598_2022_19886_MOESM1_ESM.docx]

**Supplementary Information for:**

**Identification and Characterization of a Novel Cell Binding and Cross-reactive Region on Spike Protein of SARS-CoV-2**

Hanlu Wang ^1,2,#^, Tiantian Yang^3,#^, Wenhong Jiang^2,#^, Meng Qin^1,3,#^, Ziyong Sun^4^, Wei Dai^5^, and Yongping Jiang^1,2,*^

**Supplementary Table 1. Oligonucleotides.**

| **Protein Fragment** | **Primers** |
| --- | --- |
| COVID19-SF1 | Forward: 5’-CTTGGATCC TGTGTTAATCTTACAA-3’ |
|  | Reverse: 5’-GTCTCAAGCTTATGGTGATGGTGATGATG GGATTTCAACGTACACTTT-3’ |
| COVID19-SF2 | Forward: 5’-CTTGGATCC TCCTTCACTGTA-3’ |
|  | Reverse: 5’-GTCTCAAGCTT ATGGTGATGGTGATGATG ACAAACAGTTGC-3’ |
| COVID19-SF3 | Forward: 5’-CTTGGATCC GCACCAGCAACT-3’ |
|  | Reverse: 5’-GTCTCAAGCTT ATGGTGATGGTGATGATG TTGACTAGCTACAC-3’ |
| COVID19-SF4 | Forward: 5’-CTTGGATCC GCACGTAGTGTAGCTA-3’ |
|  | Reverse: 5’-GTCTCAAGCTT ATGGTGATGGTGATGATG GATTGTACCCGCTA-3’ |
| COVID19-SF5 | Forward: 5’-CTTGGATCC GGTACAATCACTTC-3’ |
|  | Reverse: non-His tagged：5’-GTCTCAAGCTT ATCATGACAAATGG-3’ |
|  | His-tagged：5’-GTCTCAAGCTT ATGGTGATGGTGATGATG ATCATGACAAATGG-3’ |
| COVID19-SF6 | Forward: 5’- CTTGGATCC ACTTATGTCCCTGCA-3’ |
|  | Reverse: 5’-GTCTCAAGCTT ATGGTGATGGTGATGATG CATACAGCAAAGCATA-3’ |
| SARS-SF1 | Forward: 5’- CTTGGATCC GGCTCTGACCTGGACA-3’ |
|  | Reverse: 5’-GTCTCAAGCTT ATGGTGATGGTGATGATG CACAGAACATTTCAGTT-3’ |
| SARS-SF2 | Forward: 5‘-CTTGGATCC GTGGTGAGGTTTC-3’ |
|  | Reverse: 5’- GTCTCAAGCTT ATGGTGATGGTGATGATG GGCAGGGGCATT-3’ |
| SARS-SF3 | Forward: 5‘-CTTGGATCC AATGCCCCTGCCA-3’ |
|  | Reverse: 5’- GTCTCAAGCTT ATGGTGATGGTGATGATG CAGGGACACTGTGT -3’ |
| SARS-SF4 | Forward: 5‘-CTTGGATCC CTGAGGAGCACCAGCC-3’ |
|  | Reverse: 5‘-GTCTCAAGCTT ATGGTGATGGTGATGATG TGTGGCTGTGCCAGA-3’ |
| SARS-SF5 | Forward: 5‘-CTTGGATCC GGCACAGCCACAGCA-3’ |
|  | Reverse: 5‘-GTCTCAAGCTT ATGGTGATGGTGATGATG CTCATGGCAGATGGC-3’ |
| SARS-SF6 | Forward: 5‘-CTTGGATCCAGACTGGACAAGGT-3’ |
|  | Reverse: 5‘- GTCTCAAGCTT ATGGTGATGGTGATGATG ACACAGCAGGATG -3’ |


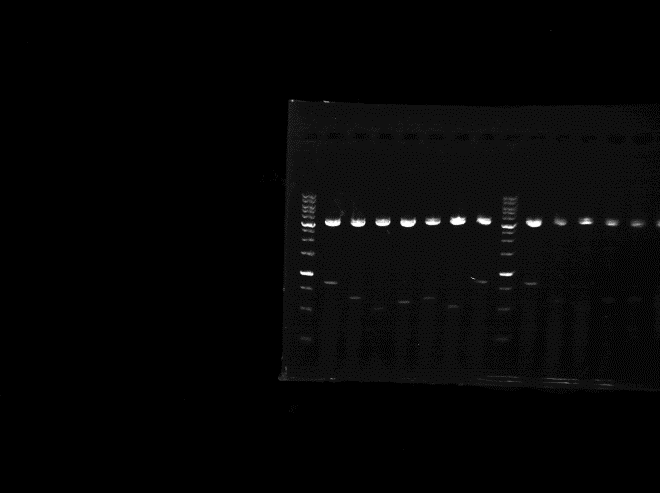


**Supplementary Figure 1**. Six protein fragments expressing plasmids of SARS-CoV-2 were constructed and verified by agarose gel electrophoresis with restriction enzymes BamH I and Hind III.


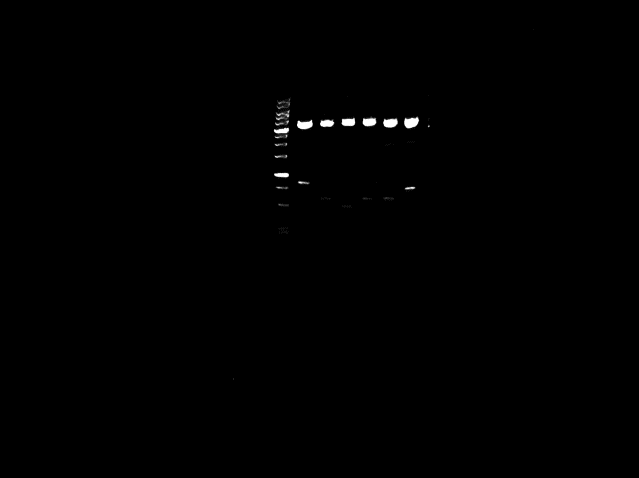


**Supplementary Figure 2**. Six protein fragments expressing plasmids of SARS-CoV were constructed and verified by agarose gel electrophoresis with restriction enzymes BamH I and Hind III.


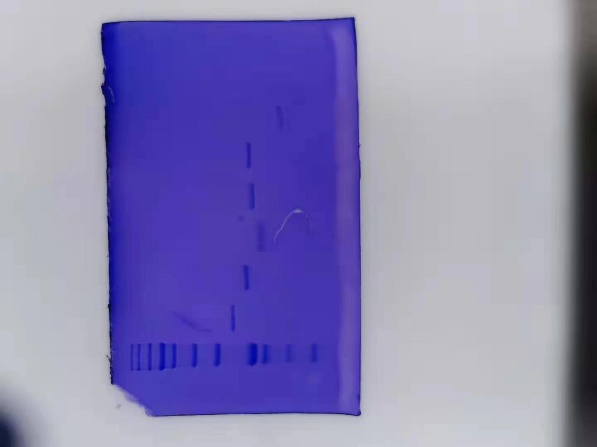


**Supplementary Figure 3**. SDS-PAGE analysis of the protein fragments expression of SARS-CoV-2.


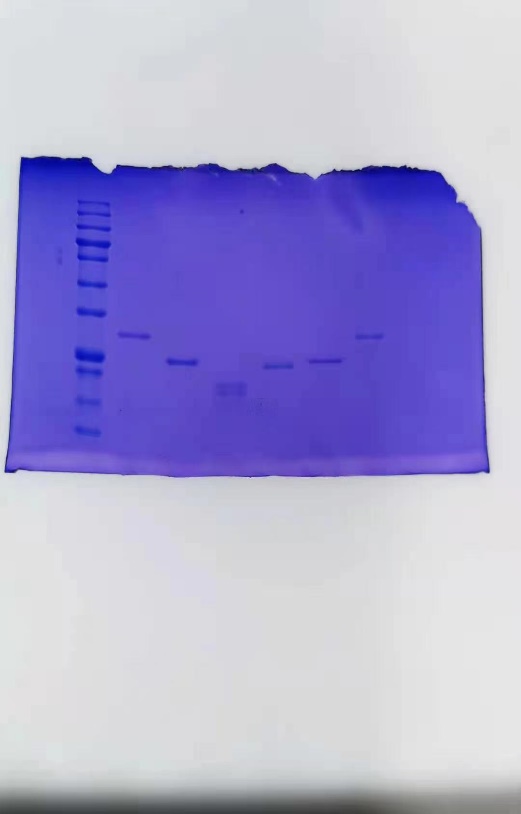


**Supplementary Figure 4**. SDS-PAGE analysis of the protein fragments expression of SARS-CoV.


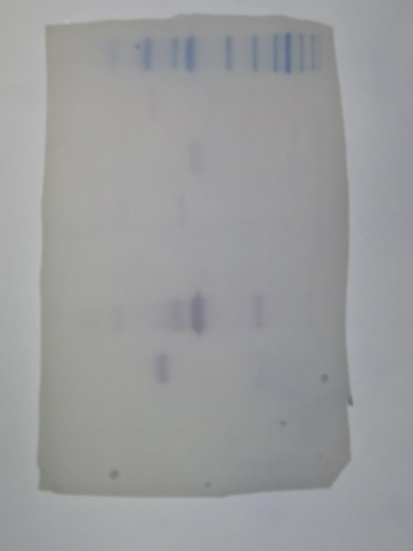


**Supplementary Figure 5**. Immunoblotting of anti-serum against COVID19-SF5 with 6 fragments of SARS-CoV-2.


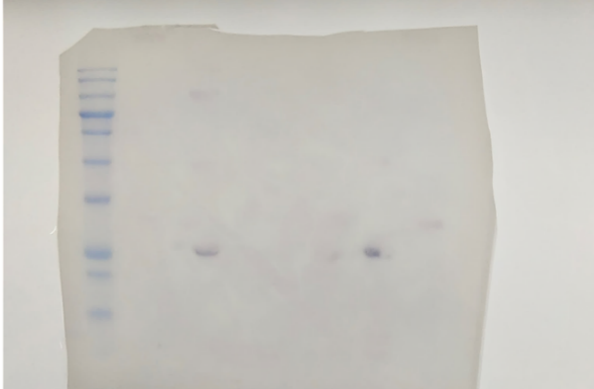


**Supplementary Figure 6**. Immunoblotting of anti-serum against COVID19-SF5 with 6 fragments of SARS-CoV.
